# Clinical candidate and genistein analogue AXP107‐11 has chemoenhancing functions in pancreatic adenocarcinoma through G protein‐coupled estrogen receptor signaling

**DOI:** 10.1002/cam4.2581

**Published:** 2019-09-30

**Authors:** Fahmi Mesmar, Bingbing Dai, Ahmed Ibrahim, Linnea Hases, Mohammed Hakim Jafferali, Jithesh Jose Augustine, Sebastian DiLorenzo, Ya'an Kang, Yang Zhao, Jing Wang, Michael Kim, Chin‐Yo Lin, Anders Berkenstam, Jason Fleming, Cecilia Williams

**Affiliations:** ^1^ Center for Nuclear Receptors and Cell Signaling Department of Biology and Biochemistry University of Houston Houston TX USA; ^2^ Department of Protein Science KTH Royal Institute of Technology Science for Life Laboratory Solna Sweden; ^3^ Department of Surgical Oncology University of Texas MD Anderson Cancer Center Houston TX USA; ^4^ Department of Biosciences and Nutrition Karolinska Institute Stockholm Sweden; ^5^ Department of Medical Sciences Uppsala University Uppsala Sweden; ^6^ National Bioinformatics Infrastructure Sweden Science for Life Laboratory Uppsala University Uppsala Sweden; ^7^ Department of Bioinformatics and Computing Science University of Texas MD Anderson Cancer Center Houston TX USA; ^8^ Axcentua Pharmaceuticals AB Stockholm Sweden; ^9^ Present address: Department of Intelligent Systems Engineering School of Informatics, Computing, and Engineering Indiana University Bloomington Bloomington IN USA; ^10^ Present address: ABK Stockholm Sweden; ^11^ Present address: Department of Gastrointestinal Oncology Moffitt Cancer Center Tampa FL USA

**Keywords:** AXP107‐11, chemoresistance, genistein, GPER1, pancreatic ductal adenocarcinoma

## Abstract

Despite advances in cancer therapeutics, pancreatic cancer remains difficult to treat and often develops resistance to chemotherapies. We have evaluated a bioavailable genistein analogue, AXP107‐11 which has completed phase Ib clinical trial, as an approach to sensitize tumor cells to chemotherapy. Using organotypic cultures of 14 patient‐derived xenografts (PDX) of pancreatic ductal adenocarcinoma, we found that addition of AXP107‐11 indeed sensitized 57% of cases to gemcitabine treatment. Results were validated using PDX models in vivo. Further, RNA‐Seq from responsive and unresponsive tumors proposed a 41‐gene treatment‐predictive signature. Functional and molecular assays were performed in cell lines and demonstrated that the effect was synergistic. Transcriptome analysis indicated activation of G‐protein‐coupled estrogen receptor (GPER1) as the main underlying mechanism of action, which was corroborated using GPER1‐selective agonists and antagonists. GPER1 expression in pancreatic tumors was indicative of survival, and our study proposes that activation of GPER1 may constitute a new avenue for pancreatic cancer therapeutics.

## Graphical abstract

A novel approach to sensitize tumor cells to chemotherapy is validated using patient‐derived pancreatic cancer models, and investigation of corresponding molecular mechanism. The clinical candidate AXP107‐11 activates the G‐coupled estrogen receptor 1 (GPER1) enabling a novel therapeutic option to improve chemoefficacy in pancreatic cancer.

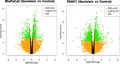

## 1 INTRODUCTION

Pancreatic ductal adenocarcinoma (PDAC) is the third‐most common cause of cancer‐related mortality in the United States. The 5‐year survival rate is a mere 8% with a median survival of six months or less.^1^ Chemotherapy with gemcitabine has been standard of care for patients with PDAC but, unfortunately, most tumors develop chemoresistance which contributes to the poor survival.^2^ Survival for resectable PDAC patients has recently been slightly improved by combining gemcitabine with capecitabine,^3^ or by combining of fluorouracil, leucovorin, irinotecan, and oxaliplatin (FORFIRINOX), as adjuvant therapy, the latter with significantly more adverse effects.^4^ Also, several major clinical trials have assessed new combinations of treatments for advanced (metastatic) PDAC for patients that failed first‐line gemcitabine treatment. Such trials include gemcitabine vs FORFIRINOX,^5^ nab‐paclitaxel in combination with gemcitabine,^6^ and nanoliposomal irinotecan in combination with 5‐FU/leucovorin.^7^ However, the need for safe, affordable and less toxic drugs to augment the efficacy of chemotherapies remain indispensable.

Gemcitabine exerts its anti‐tumorigenic effect by interfering with DNA replication, thereby activating cell apoptosis.^2^ This activity requires that gemcitabine is transported into the cell and metabolically converted to active di‐ and triphosphate gemcitabine by various kinases. The underlying mechanism of chemoresistance is not fully understood, but gemcitabine can become inactive through deamination and dephosphorylation.^2^ Consequently, deregulation of gemcitabine transporters or metabolic enzymes are potential chemoresistance mechanisms. Other pathways, such as activation of NFκB signaling,^8^ and acquisition of mesenchymal phenotype and stem cell‐like features, have also been implicated in the chemoresistance of PDAC.

Genistein is a phytoestrogen that has been shown to augment the antineoplastic effect of different chemotherapeutic drugs, including gemcitabine in pancreatic cancer cell lines and xenografts.^9^, ^10^, ^11^ Genistein is considered safe, but has a poor oral bioavailability. A clinical phase I trial with a patented bioavailable crystalline form of genistein, AXP107‐11,^12^ added to gemcitabine regimen, resulted in increased serum levels of AXP107‐11 and did not indicate significant toxicity for PDAC patients.^13^ Thus, this may be an avenue to enhance therapeutic efficacy. As a phytoestrogen, genistein can bind and modulate the activities of the nuclear estrogen receptors ERα (ESR1) and ERβ (ESR2), and can also activate the G protein‐coupled estrogen receptor 1 (GPER1/GPR30). Other, non‐ER pathways, such as NFκB, MAPK/ERK, and PI3K/Akt/mTOR^14^, ^15^ and apoptosis signaling (reviewed in Ref. 16) have been linked to genistein activity in different types of tumor cells. The precise molecular mechanism of genistein (or AXP107‐11) in PDAC is, however, unknown. Further, we lack an understanding to predict which individual PDAC patients may respond to such therapy.

Here, we have conducted patient‐derived xenograft (PDX) PDAC model experiments, which take the genetic and cellular heterogeneity of the clinical disease into account. We have explored the efficacy of AXP107‐11 co‐treatment, demonstrating a chemoenhancing effect in 8 out of 14 PDX tumors ex vivo, and validated this effect in vivo. We also propose a treatment‐predictive PDAC gene expression signature. In order to provide a comprehensive understanding of the molecular mechanism of action of genistein and AXP107‐11 in PDAC, we performed gene expression profiling using RNA sequencing (RNA‐Seq) and functional and mechanistic experiments in cell lines. Based on this data, we suggest that the chemoenhancing mechanism of action include activation of GPER1, that mediates a synergistic effect when combined with gemcitabine.

## 2 MATERIALS AND METHODS

### 2.1 Cell cultures and treatments

MiaPaCa2 and PANC1 were cultured in DMEM containing 4.5 mg/mL glucose and L‐glutamine and 10% fetal bovine serum. Cell lines were tested regularly for *Mycoplasma* and cell authentication was performed. Cells were treated with indicated concentrations of gemcitabine, genistein, GPER1 agonist G1 (Tocris Bioscience), GPER1 antagonist G15 (Tocris Bioscience), or AXP107‐11 (Axcentua Pharmaceuticals AB). All of the chemicals except gemcitabine were prepared in DMSO and the final concentration of DMSO did not exceed 0.1%. Negative control cell lines HEL and THP1 were cultured in RPMI‐1640 medium with 10% FBS and 1% penicillin‐streptomycin, and HepG2 in EMEM media with 10% FBS, 1% NEAA and 1% L‐Glut.

### 2.2 Cell proliferation assay

MTS reagent (3‐(4,5‐dimethylthiazol‐2‐yl)‐5‐(3‐carboxymethoxyphenyl)‐2‐(4‐sulfophenyl)‐2H‐tetrazolium, PES: phenazine ethosulfate) was used to measure cell proliferation. For each cell line, approximately 3000 cells were plated in quintuple fashions in 96‐well plates. After 24 hours, cells were treated for indicated times (24‐96 hours) and cell proliferation measured by adding 20 μL of MTS reagent to 100 μL of media followed by incubation for 2 hours at 37°C and 5% CO_2_. Absorbance was measured at 490 nm using SpectraMax spectrophotometer. Significance was determined by unpaired two‐tailed *t* test. The IC_50_ was measured with the log (inhibitor) vs normalized response, variable slope and nonlinear regression fitting curve in Graphpad Prism. We determined the combination index (CI) to differentiate between additive and synergistic effects (antagonism CI > 1; additive CI = 1; synergism CI < 1) and visualized this in isobolograms.

### 2.3 RNA isolation, cDNA synthesis and qPCR analysis

RNA was isolated using Trizol/Chloroform extraction and purified on RNeasy Qiagen kit (Qiagen). Concentration and quality was measured using NanoDrop and Tape Station (Agilent). cDNA was prepared from 1 μg of RNA using iScript cDNA synthesis kit (Biorad), and a combination of oligo(dT) and random primers. iTaq SYBR Green supermix kit was used for PCR amplification. Relative gene expression was measured using ΔΔC_T_ method, with 18S and GAPDH as reference genes.

### 2.4 RNA‐Seq analysis

Poly‐A library preparation and RNA‐Seq using Illumina HiSeq rapid mode was performed at Sweden's National Genomics Infrastructure (NGI). At least 15 million single reads (50 bp) were generated for each sample, and mapped against human genome (GRCh37) using Tophat/2.0.4. Reads with multiple alignments were removed by using picard‐tools/1.29, htseq/0.6.1 was used to count reads per transcript, and cufflinks/2.1.1 to normalize read count to transcript length and total number of the reads per sample (Fragments Per Kilobase per Million, FPKM). The Limma‐Voom method was used to calculate differential gene expression and corresponding fold change (FC), *P*‐value, and false discovery rate (FDR) adjusted *P*‐value. The Benjamini Hochberg method was used to adjust for multiple hypothesis testing. Genes were denoted as significantly differentially expressed when FDR ≤0.05, log_2_FC ≥|0.4|, and FPKM (treated) >1. Enriched sub‐networks and gene ontologies/biological functions were identified using Pathway Studio's Expression regulatory sub‐network enrichment, Elsevier's Pathway Studio (11.2.5.9) (https://www.elsevier.com/solutions/pathway-studio-biological-research). Two biological replicates were analyzed for each condition. Data is uploaded under GEO accession number [http://www.ncbi.nlm.nih.gov/geo/query/acc.cgi?acc=GSE97766].

### 2.5 Western blots

Protein crude extracts were prepared using RIPA buffer (Thermo Fisher Scientific) supplemented with Roche protease‐phosphatase inhibitors cocktail (Sigma Aldrich). Protein was quantified by using RC‐DC protein assay kits (Bio‐Rad), and 30 μg of total protein were electrophoresed in 10% polyacrylamide gel (Bio‐Rad). Proteins were transferred to PVDF membrane, blocked in 5% nonfat milk TBST buffer for 1 hours at room temperature, and incubated with anti‐GPER1 (Thermo Fisher Scientific, PA5‐33691, lot TK2663642, rabbit, 1:500 dilution), anti‐PARP (Cell Signaling Technology, #9542S, lot:14, 1:1000 dilution), anti‐caspase 3 (Cell Signaling Technology, #9662S, lot:18, 1:1000 dilution), anti‐cleaved‐caspase 3 (Cell Signaling Technology, #9661S, lot:43, 1:1000 dilution), anti‐caspase 9 (Abcam, ab202068, lot GR305416‐9, 1:2000 dilution), anti‐MUC1 (Cell Signaling Technology, #4538, lot: 6, 1:1000 dilution), anti‐β‐actin, (Abcam, ab8226, lot: GR3249122‐1, 1:3000 dilution) or anti‐GAPDH antibody (Thermo Fisher Scientific, #MA5‐15738, lot RF230546A, 1:10,000 dilution) in 1% nonfat milk TBST overnight at 4°C. After washing, protein bands were detected by using species‐specific secondary antibodies conjugated with horseradish peroxidase (Cell Signaling Technology, anti‐rabbit IgG #7074S, lot26, and anti‐mouse IgG # 7076S, lot 32, 1:10,000 dilutions) for 2 hours at room temperature, then incubated with the substrate (Bio‐Rad). For GPER1 detection, the PA5‐33691 antibody was validated using recombinant protein extract as positive control, and the erythroleukemia HEL and leukemic monocyte THP1 cell lines (that have no mRNA evidence of GPER1 per the http://www.proteinatlas.org) as negative controls.

### 2.6 Apoptosis assays

Annexin V and DNA staining were used to evaluate cell apoptosis during G1 treatment (2 µmol/L for 48 hours). Annexin V was detected by FITC and propidium iodide was used for nucleic acid staining. BD LSRFortessa analyzer was used to detect the fluorescence, and FlowJo (FlowJo, LLC) was used for data analysis.

### 2.7 The Cancer Genome Atlas expression and survival data

The Cancer Genome Atlas (TCGA) expression (RSEM and FPKM) and overall survival data was analyzed for 176 pancreatic cancer patients using OncoLnc 17 and the Human Protein Atlas.^18^, ^19^ The median GPER1 mRNA expression in the data set was 2.3 FPKM (or 78 RSEM), and 64% of patients had levels indicative of expression at functional level (>1 FPKM; or RSEM >65), whereas expression below 1 FPKM was considered nonexpressed. We compared the higher 64% to the lower 36% (Cut‐off 65 RSEM), as well as the 36% low vs 36% high (using <65 RSEM and >96 RSEM cut‐off). Logrank *P*‐value <.05 was considered significant.

### 2.8 Patient‐derived xenograft model

PDX tumors were established from PDAC patients at MD Anderson Cancer Center with written informed consent for our IRB‐approved laboratory research protocol (LAB07‐0854). The detailed method has been reported in our previous publication.^20^ Patient clinical characteristics are provided in Table S1A.

### 2.9 Live tissue sensitivity assay

Cryopreserved PDAC PDX were recovered from storage medium and implanted in nude mice with previously published method.^20^ At size 1.5 cm in diameter, tumors were harvested and cut into ~200 µm thick slices using the Krumdieck Tissue Slicer (Alabama Research & Development), and cultured in 96‐well plate as previously described.^21^ Suitable concentrations of AXP107‐11 treatments were calibrated in a pilot experiment, and concentrations of gemcitabine had been determined in previous studies.^21^ Tissue slices were treated with vehicle (DMSO), AXP107‐11, gemcitabine, alone or in combinations for 72 hours at indicated doses. Viabilities were measured with PrestoBlue as described previously,^21^ and fluorescence values were normalized by the values of nontreatment control tissue slices or to gemcitabine only treatment, as indicated. Student's *t* test (two‐tailed) was used to test for significance.

### 2.10 In vivo PDX model

Tumors PATX179, PATX53, and PATX55 were implanted in female nude (BALB/c) mice as previously described.^20^ When tumors reached ~100 mm^3^, mice were randomly divided into 4 groups with 5 mice in each group. Mice were treated for 3 weeks through intraperitoneal (ip) injection with gemcitabine (50 mg/kg body weight, twice a week), AXP107‐11 (10 mg/kg body weight, daily), or the combination of both agents, and the fourth group was treated with vehicle only. AXP107‐11 and gemcitabine were both formulated with phosphate‐buffered saline (PBS). One month after the treatment, all mice were sacrificed. One‐way ANOVA test was used to compare the different treatment groups (Graphpad 6.0).

### 2.11 Biomarker analysis

RNA‐Seq of 3 resistant (PATX148, PATX60 and PATX112) and 4 sensitive (PATX53, PATX55, PATX39 and PATX50) PDXs was performed at the Institution of Applied Cancer Science of MD Anderson Cancer Center. Correlation between RNA‐Seq data and the treatment responses were analyzed using R program packages. Negative binomial models for each gene were built and Wald tests for the differential expression were analyzed. DESeq2 was used to normalize the data and compute the log_2_ FC with shrinkage, and Benjamini Hochberg method was used to adjust for multiple hypothesis testing and to assess the FDR. Significant genes were selected with FDR <0.05 and log_2_ FC >|2|.

## 3 RESULTS

### 3.1 AXP107‐11 has synergistic effect when combined with gemcitabine

**Figure 1:**
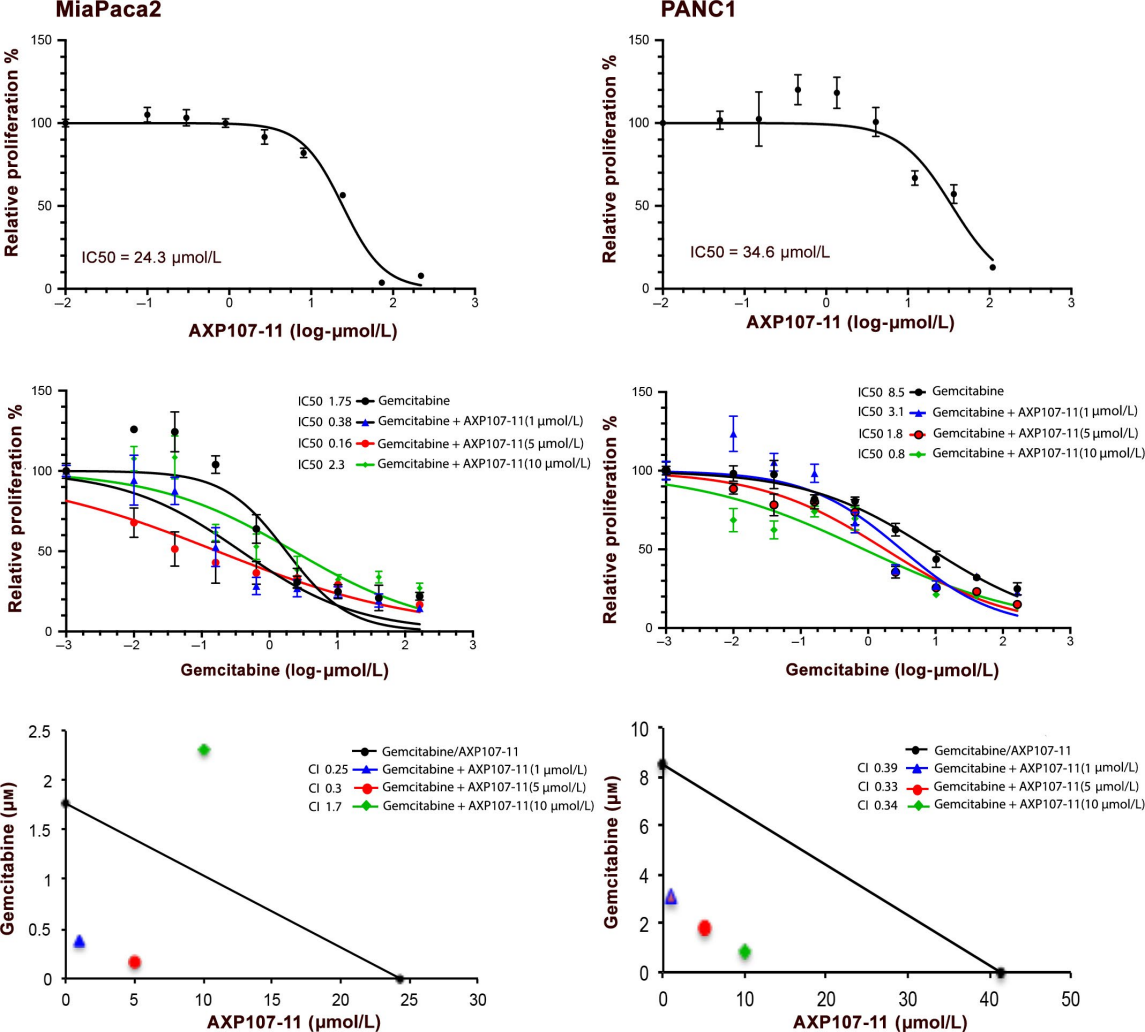
Cell viability dose‐response curves of AXP107‐11 and test of synergistic interaction with gemcitabine. MiaPaCa2 (left) and PANC1 (right) were treated with increasing concentrations of AXP107‐11 (0.01‐200 μmol/L, top panels) or gemcitabine (0.001‐200 μmol/L, middle panels) in order to determine respective IC_50_. IC_50_ was measured with the log (inhibitor) vs normalized response‐variable slope and nonlinear regression fitting curve in Graphpad Prism. Increasing concentrations of gemcitabine in combination with three different doses of AXP107‐11 (1, 5, and 10 μmol/L, middle panels) were tested to calculate the IC_50_ values of the combinations and combination index (CI). Isobolograms graphically visualize whether the combinations result in antagonistic (CI > 1), additive (CI = 1), or synergistic (CI < 1) effects, in respective cell line (lower panels)

The clinical candidate AXP107‐11 (crystalline genistein sodium salt dehydrate) has been developed to improve the bioavailability of genistein.^12^, ^13^ AXP107‐11 is thus a form of genistein, but the precise chemoenhancing effect of the AXP107‐11 compound itself has not been characterized. In order to define this, we first tested its antiproliferative function in vitro using two different PDAC cell lines (MiaPaCa2 and PANC1) that both classify as the poor‐survival quasimesenchymal (QM) PDAC subtype.^22^ We measured the half maximal inhibitory concentration (IC_50_) for AXP107‐11 and gemcitabine monotherapy, respectively. We found that the IC_50_ of AXP107‐11 monotherapy was 24.3 µmol/L for MiaPaCa2, and 34.6 µmol/L for PANC1 cells (Figure 1, top panels). Corresponding IC_50_ values for gemcitabine monotherapy was 1.8 µmol/L and 8.5 µmol/L, respectively (Figure 1, middle panels). Next, we tested whether AXP107‐11 could enhance the effect of gemcitabine. We found a dose‐dependent chemoenhancing effect (Figure 1, middle panels). By adding 1 µmol/L AXP107‐11, the IC_50_ for gemcitabine was reduced from 1.76 µmol/L to 380 nmol/L in MiaPaCa2. Addition of 5 µmol/L AXP107‐11 further reduced the IC_50_ to 160 nmol/L. In the more gemcitabine‐resistant PANC1 cells, addition of 1µmol/L AXP107‐11 reduced the IC_50_ from 8.5 µmol/L (gemcitabine only) to 3.1 µmol/L, and increasing concentrations of AXP107‐11 (5 µmol/L and 10 µmol/L) lowered the IC_50_ to 1.8 µmol/L and 86 nmol/L, respectively. To determine whether the noted combinatory effects were additive or synergistic, we calculated the combination index (CI). A CI larger than 1 represents an antagonistic effect, if CI equals 1 the effect is additive, and if CI is less than 1 the effect is synergistic. The isobolograms in Figure 1 (lower panels) graphically illustrate that all tested AXP107‐11 concentrations (1, 5 and 10 µmol/L) had synergistic effects when combined with gemcitabine in PANC1 (Figure 1, right panel). Similarly, in MiaPaCa2, both 1 µmol/L and 5 µmol/L had synergistic effects, but not 10 µmol/L (Figure 1, lower left panel). We conclude that in the tested cell lines, addition of AXP107‐11 to gemcitabine treatment produced a synergistic antiproliferative effect.

### 3.2 AXP107‐11 has chemoenhancing property in patient‐derived PDAC xenografts ex vivo and in vivo

**Figure 2:**
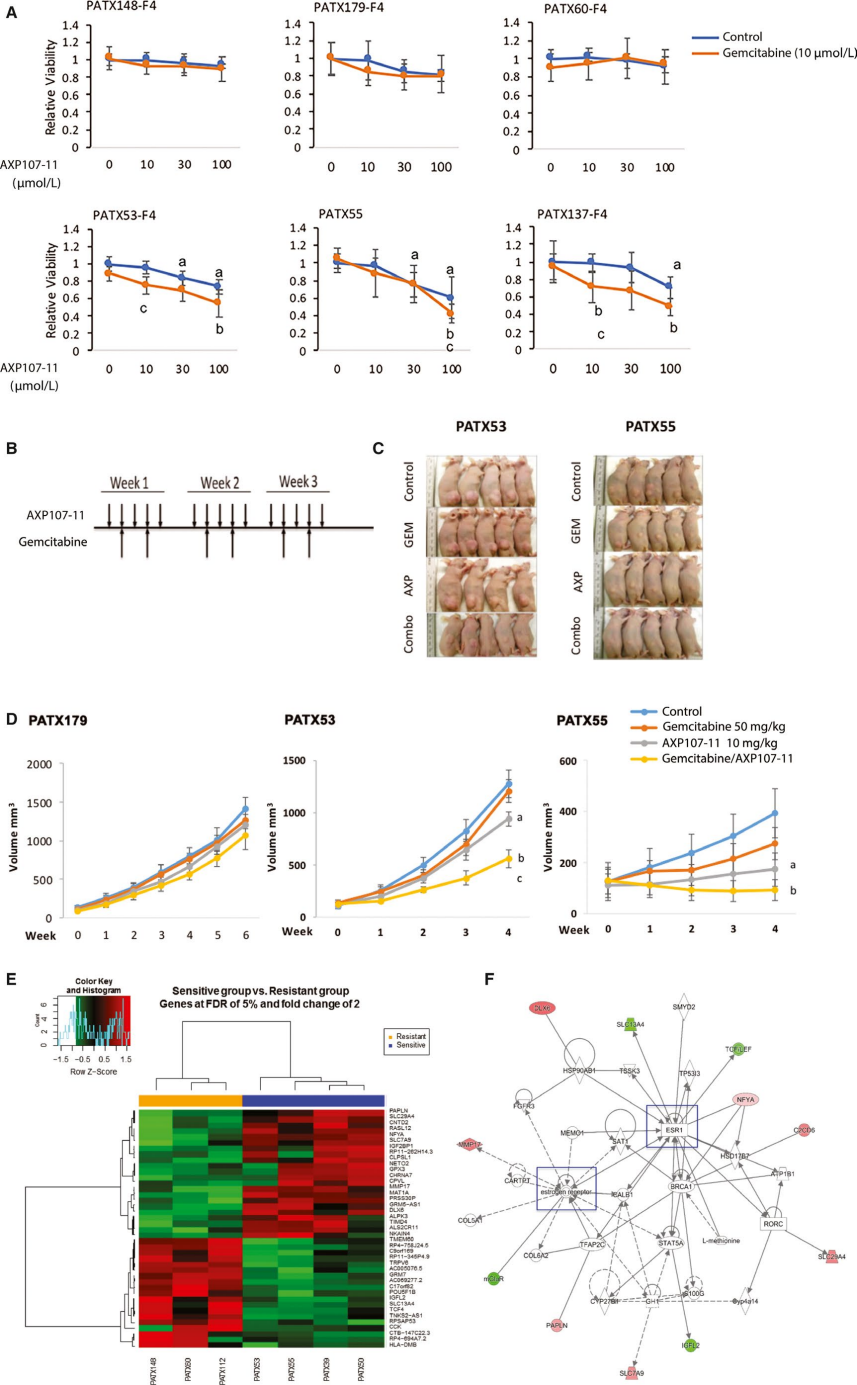
Ex vivo and in vivo validation of AXP107‐11 and gemcitabine co‐treatment in PDAC PDXs. A, Representative results from ex vivo analysis of AXP107‐11 nonresponders (top) and responders (bottom). Tissue slices from PDAC PDXs were treated with vehicle (Control) alone (blue dot at AXP107‐11 dose 0), gemcitabine (GEM, 10 µmol/L) alone (orange dot at AXP107‐11 dose 0), with AXP107‐11 alone (blue line at 10, 30 or 100 μmol/L), and their combinations (orange line at 10, 30 or 100 μmol/L). Cell viability was measured after 72 h (PrestoBlue). Significance was analyzed using unpaired, two‐tailed *t* test. Tumors were identified as sensitive if viability was reduced >40% with *P* < .05. No PDXs responded significantly to gemcitabine monotreatment (orange vs blue dot at 0 μmol/L AXP107‐11). [a] indicates significance of AXP107‐11 monotreatment (blue line at 10, 30 or 100 μmol/L) compared to vehicle control (blue dot at dose 0), [b] co‐treatment (orange line at 10, 30 or 100 μmol/L) compared to gemcitabine only (orange dot at AXP107‐11 0 µmol/L), and [c] co‐treatment (orange line at 10, 30 or 100 μmol/L) compared to AXP107‐11 only (blue line at 10, 30 or 100 μmol/L) for respective concentration. B, PDXs from PATX179 (resistant tumor), PATX53 (responsive), and PATX55 (responsive) were established in nude mice. Mice were treated with PBS, AXP107‐11 (10 mg/kg, ip) 5 d/wk, gemcitabine (GEM, 50 mg/kg, ip) twice a week, or the combination of both agents, for 3 wk. C, Tumors of two sensitive PDX models at the experiment's end point (AXP indicates AXP107‐11). D, Tumor volumes were measured weekly and significance between different treatment groups was tested using one‐way ANOVA. One resistant PDX tumor (PATX179, left), and two responsive PDXs (PATX53 and PATX55, middle and right). E, Heatmap analysis indicates 41 differentially expressed genes between sensitive and resistant PDAC PDXs. F, Ingenuity Pathway Analysis of predictive PDX signature indicates an enriched gene network associated with estrogen signaling

In order to corroborate the chemoenhancing effect of AXP107‐11 and explore its efficacy in clinical material, we used ex vivo culture of PDX PDAC models.^21^ This ex vivo platform has been shown to predict the clinical response to anticancer drugs in PDAC patients with resectable disease.^21^ We compared the efficacy of combinatory treatment to that of either treatment alone, or to vehicle. PDX tumors originating from 14 different PDAC patients (patient characteristics in Table S1A) were grown as slice cultures and treated with gemcitabine alone (10 µmol/L, dosage based on previous study of borderline gemcitabine response in PDX PDAC tumors^21^), AXP107‐11 alone (three doses tested: 10, 30 and 100 µmol/L), combinations, or vehicle. These tumors are generally resistant to gemcitabine treatment and, as shown in Table 1, none of the 14 tumors responded to 10 µmol/L gemcitabine in the ex vivo slice culture. Using previously defined criteria (reduction of viability of 40% or more, and *P* < .05), we found that adding AXP107‐11 to the gemcitabine treatment rendered 8 out of the tested 14 PDXs (57%) sensitive to treatment (Table 1). Representative results of responders and nonresponders are illustrated in Figure 2A, and complete results are available in Figure S1 and Table S2. While two tumors responded to AXP107‐11 monotreatment at the highest dose (PATX55, Figure 2A blue line and Table 1, complete p‐values provided in Table S2; and PATX173, Figure S1), there was a considerably larger response to combinatory treatment. Our data show that adding AXP107‐11 to gemcitabine was significantly more effective than gemcitabine alone, or AXP107‐11 alone, in a large subset of gemcitabine‐resistant PDXs.

**Table 1:** Treatment efficacy measured by live tissue sensitivity assay of PDX tissue ex vivo

	Viability					*P* value	
	DMSO	GEM	AXP107‐11	APX107‐11 + GEM	Subtype	Combination vs DMSO	Combination vs GEM
PATX60	1	0.90	0.91	0.94	C	.8	.4
PATX148	1	0.89	1.02	0.89	QM	.21	.2
PATX179	1	0.99	0.81	0.80	QM	.06	.1
PATX112	1	0.94	0.81	0.78	C	.002^a^	.02^b^
PATX162	1	0.89	0.72	*0.60*		.03^a^	.08
PATX39	1	0.76	0.63	*0.60*	C	.0005^a^	.2
PATX193*	1	0.93	*0.55*	*0.51*		.003^a^	*.02* ^b^
PATX176*	1	1.03	0.71	*0.55*	EX	.01^a^	*.01* ^b^
PATX53*	1	0.89	0.74	*0.55*	QM/MET	.002^a^	*.008* ^b^
PATX137*	1	0.95	0.71	*0.48*	C	.02^a^	*.004* ^b^
PATX173*	1	1.08	0.64	*0.55*		.01^a^	*.003* ^b^
PATX213*	1	0.96	0.68	*0.51*	MET	.007^a^	*.003* ^b^
PATX50*	1	0.91	0.66	*0.50*	C	.002^a^	*.0008* ^b^
PATX55*	1	1.05	*0.60*	*0.42*	C	.0003^a^	*.0002* ^b^

Note

PDAC PDX tumors were harvested, cut into slices, and cultured in 96‐well plates. Tissue slices were treated with vehicle (DMSO), AXP107‐11 (100 µmol/L), gemcitabine only (GEM, 10 µmol/L), or combinations for 72 h. Viabilities were measured using PrestoBlue, and fluorescence values were normalized to nontreatment control tissue slices (DMSO). A reduction in viability of at least 40% (0.60, *italics*) was considered as efficacious. Significance were calculated using Student's *t* test (two‐tailed) comparing co‐treatment with ^a^vehicle (DMSO) or ^b^gemcitabine only treatment (GEM), as indicated. No tumors responded significantly to GEM treatment (gemcitabine resistant).

* Tumor that fulfill the criteria viability max 60% of control (0.60) and viability reduction *P* < .05 upon combinatory treatment compared to GEM treatment.

In order to confirm the efficacy of combinatory treatment in vivo*,* selected tumors identified in the ex vivo culture as responsive (primary tumor PATX55 and liver metastasis PATX53) or resistant (liver metastasis PATX179) were established in vivo. Implanted mice were treated with AXP107‐11 and gemcitabine, alone or in combination (Figure 2B,C) and the growth of tumors were measured. The PDX tumor that was resistant in the ex vivo analysis, was resistant also in vivo (PATX179, Figure 2D, left panel) while the two tumors indicated as sensitive in the ex vivo analysis, PATX53 and PATX55, demonstrated significantly reduced tumor growth upon combination treatment also in vivo (Figure 2C,D). We note that the tumor which responded to AXP107‐11 monotherapy ex vivo, PATX55, did so also in vivo, but co‐treatment had a considerably stronger effect and inhibited tumor growth completely in vivo (even reduced the volume of the tumors). Further, treatment of PATX53 clearly showed that neither gemcitabine nor AXP107‐11 monotreatment had much efficacy ex vivo nor in vivo, while the combination did. Thus, both the ex vivo and in vivo assays demonstrated that AXP107‐11 sensitized a subset of PDAC PDXs to gemcitabine treatment, and that this combinatory treatment was superior compared to each type of single treatment.

To explore whether a gene signature could predict which tumors may respond favorably, we analyzed the correlation between the tumors’ transcriptional profile (RNA‐Seq) pre‐treatment and its response to the combination therapy. Comparing 4 sensitive tumors and 3 resistant tumors, we identified 41 differentially expressed genes that could separate the two groups (Figure 2E and Table S1B). This signature included *MMP17, DLX6*, and *CLPSL1* which were expressed 8‐ to 24‐fold more in responsive tumors. We used the Ingenuity platform to explore related pathways, which revealed that 11 of the 41 genes were connected to estrogen signaling (Figure 2F). Additionally, we noted connections (visualized in Figure S3) to NFκB, Vitamin D receptor (VDR), Ca^2+^ release, and cholinergic receptor nicotinic alpha 7 (CHRNA7). Overall, we demonstrate that adding AXP107‐11 to gemcitabine treatment can enhance treatment efficacy in a subset of tumors and we propose a treatment‐predictive profile.

### 3.3 Gene expression profiling identifies gemcitabine‐mediated genome‐wide impacts

**Figure 3:**
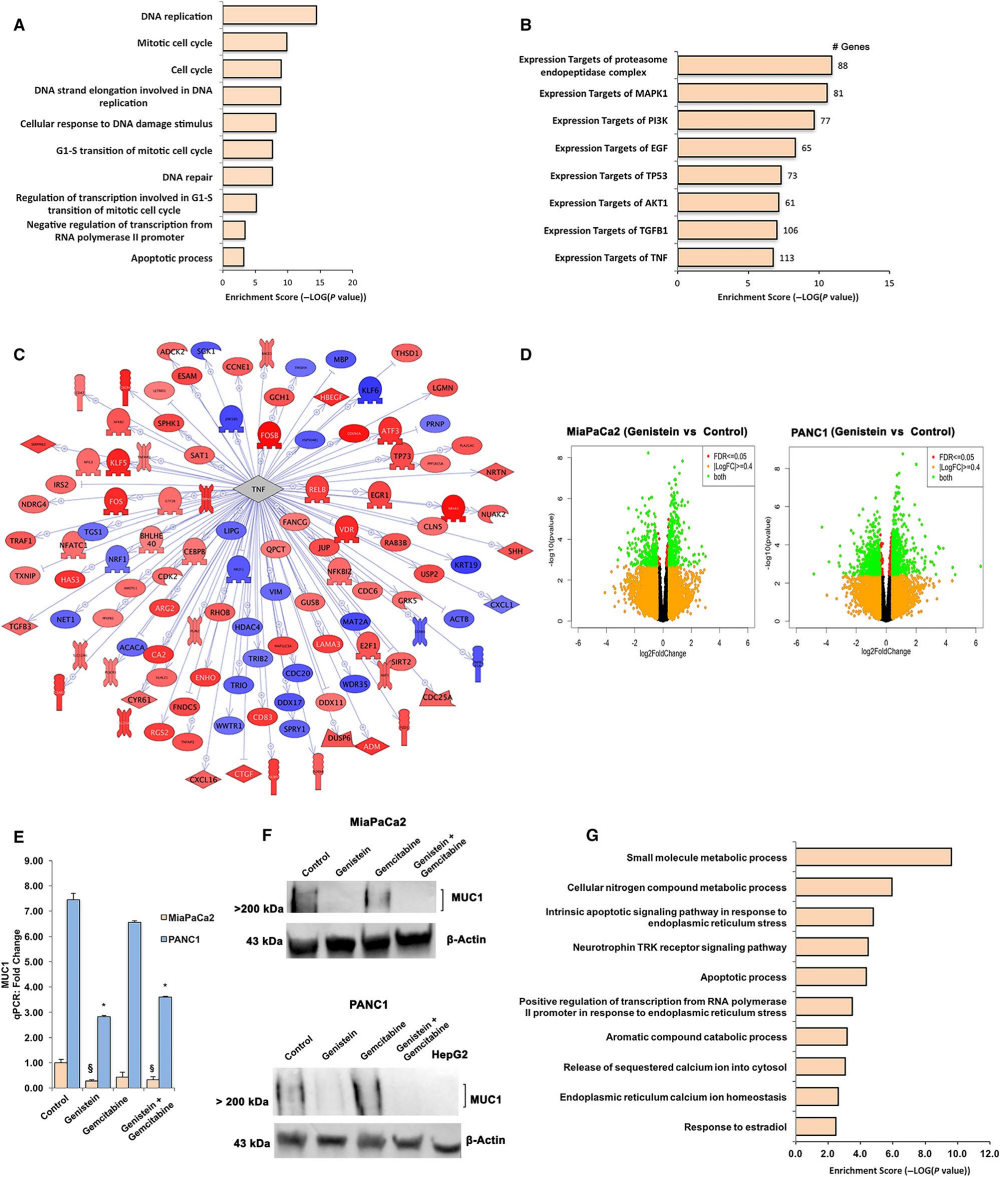
Transcriptomic effects of gemcitabine and genistein treatment in MiaPaCa2 and PANC1. RNA‐Seq was performed and 834 genes was determined as regulated by gemcitabine in both cell lines. Among these genes, several biological functions (A) and subnetworks (B) were enriched, including (C) targets of TNFα‐signaling (n = 113). D, RNA‐Seq determined genes regulated by genistein, as depicted by Volcano plots. E, qPCR confirms RNA‐seq data of increased MUC1 expression in chemoresistant PANC1 compared to MiaPaCa2, and subsequent repression by genistein treatment (24 h) in both cell lines. F, Western blot corroborates MUC1 protein expression in MiaPaCa2 and PANC1 and the subsequent repression by genistein treatment (48 h) in both cell lines. Protein above 200 kDa is indicated, as expected. The HepG2 cell line have no mRNA evidence of MUC1 (per the http://www.proteinatlas.org) and was used as negative control, β‐actin was used as a loading control. G, Upon addition of genistein to gemcitabine treatment, 154 genes were regulated in both cell lines, and the biological functions enriched among these genes are visualized. X‐axes in A, B and F show the enrichment score [−log_10_(*P* value)]

The mechanism of action for the noted chemoenhancing effect of AXP107‐11, or genistein, remains undefined. To explore this at the molecular level, we used the cell lines MiaPaCa2 and PANC1 and RNA‐Seq. We first compared the two cell lines, and investigated the response that gemcitabine alone induced. Approximately 15 000 genes were detected as expressed above cutoff (>1 FPKM) in either cell line, with a large overlap (85%) of expressed genes (Figure S2A). Notable differences between the two cell lines included regulators of G protein signaling and calcium channels (Table S3 lists the top‐50 differentially expressed genes for each cell line). Genes related to drug resistance (*MUC1, RRM1, TM4SF1, ABCC1, ABCC3, HMGA2* and long noncoding RNA *HOTAIR*) were upregulated in the more gemcitabine‐resistant PANC1 compared to MiaPaCa2 (data not shown). Next, we identified the genes that were modified by gemcitabine monotreatment. We chose a low concentration of gemcitabine (40 nmol/L, 24 hours) which did not induce extensive level of cell death (Figure 1, middle panels) in order to enable the identification of molecular mechanisms and chemoenhancing effects. We found that 834 genes were regulated by gemcitabine in both cell lines (Figure S2B, top‐50 genes for each cell line are provided in Table S4). Gene ontology classifications indicated a significant enrichment for genes related to DNA replication, mitotic cell cycle, DNA repair, and apoptosis (Figure 3A). Gemcitabine is known to induce apoptosis by interfering with DNA synthesis, causing DNA damage and reducing mitosis,^2^ and this mechanism was thus readily detected at the transcriptome level in our data. Subnetwork analysis identified that MAPK1, PI3K/Akt, TGFβ1, and NFκB/TNFα‐regulated genes were significantly overrepresented upon gemcitabine treatment (Figure 3B,C). The high number of TNFα‐regulated genes highlights the central role of the TNFα‐mediated pathway in gemcitabine response. TNFα activates NFκB transcription, and members of the NFκB transcription complex were also significantly upregulated upon gemcitabine treatment in both cell lines (including *RELB* and *NFKB2*, Figure S2C). Thus, we here describe the full genome‐wide response to gemcitabine in PANC1 and MiaPaCa2, including the specificities of each cell line.

### 3.4 Chemoenhancing pathways of genistein include intrinsic apoptosis and MUC1 repression

The main difference between AXP107‐11 and genistein is the bioavailability. Once inside the cells, such as in cell line treatments, the molecules are expected to behave in identical manners. To explore how the addition of genistein (50 μmol/L, 24 hours) modified the transcriptome. We corroborated that genistein, like AXP107‐11, significantly decreased cell proliferation with corresponding IC_50_ (Figure S2D,E, left panels), and similar synergistic effects (Figure S2D,E, middle and right panels). Genistein alone significantly regulated genes in both cell lines (Figure 3D, Table S5 for top‐50 regulated genes) and 148 of these gene regulations were common for both cell lines (Figure S2F). Functional annotation of these genes revealed an enrichment for the biological processes calcium ion release, intrinsic apoptotic signaling in response to endoplasmic reticulum stress (eg *DDIT3* and *ITPR1*), and estradiol signaling (Figure S2G), which are particularly relevant to genistein's proposed functions.^15^ A different estrogenic ligand (bisphenol A) has been found to impact Ca^2+^ in mouse pancreatic beta cells,^23^ and DDIT3 which was significantly upregulated in both cell lines (Figure S2H) is a marker of endoplasmic reticulum stress which can activate apoptosis.^24^ We also note that the nuclear receptor VDR was regulated in both cell lines, and NR4A1 (also known as Nur77 or NGFIB) in MiaPaCa2. NR4A1 has been implicated in oncogenesis and tumor suppression, as well as with endoplasmic reticulum stress, in PDAC cells.^25^

Next, to detail the chemoenhancing property of genistein, we compared RNA‐Seq between co‐treatment and gemcitabine monotherapy (top‐50 genes listed in Table S6) and found that 154 genes were significantly modulated by this in both cell lines (Figure S2I). Co‐treatment modified genes involved in intrinsic apoptosis in response to endoplasmic reticulum stress, release of sequestered calcium ion into cytosol, and response to estradiol (Figure 3G). MUC1, which mediates gemcitabine resistance in pancreatic cancer,^26^, ^27^ was repressed upon addition of genistein in both cell lines (Figure S2H) and this was corroborated using qPCR (Figure 3E) and western blot (Figure 3F). Collectively, our data provide a genome‐wide map of how genistein potentiates the anti‐tumor functions of gemcitabine.

### 3.5 GPER1 is a potential mediator of genistein activity in pancreatic cancer cells

In order to decipher the molecular mechanism whereby genistein sensitizes PDAC towards gemcitabine treatment, we reviewed the RNA‐Seq data. Estradiol signaling was an overrepresented function among regulated genes, and was also indicated in the treatment‐predictive profile of the PDX tumors above (Figure 2F). Other connections of the predictive profile (visualized in Figure S3) such as NFκB, VDR, Ca^2+^ release, and cholinergic receptor nicotinic alpha 7 (CHRNA7), were also regulated by genistein in vitro, and have been linked to genistein treatment previously. While genistein is a known phytoestrogen, there is no clear evidence at neither mRNA nor protein level that the nuclear estrogen receptors ERα or ERβ are expressed in the pancreas or PDAC.^28^ In our data set for the PANC1 and MiaPaCa2 cell lines, mRNA for ERα and ERβ was not detectable by RNA‐Seq (FPKM <0.1 for both). Thus, we excluded their involvement and considered the membrane‐bound GPER1 as a potential target for estrogenic signaling.

**Figure 4:**
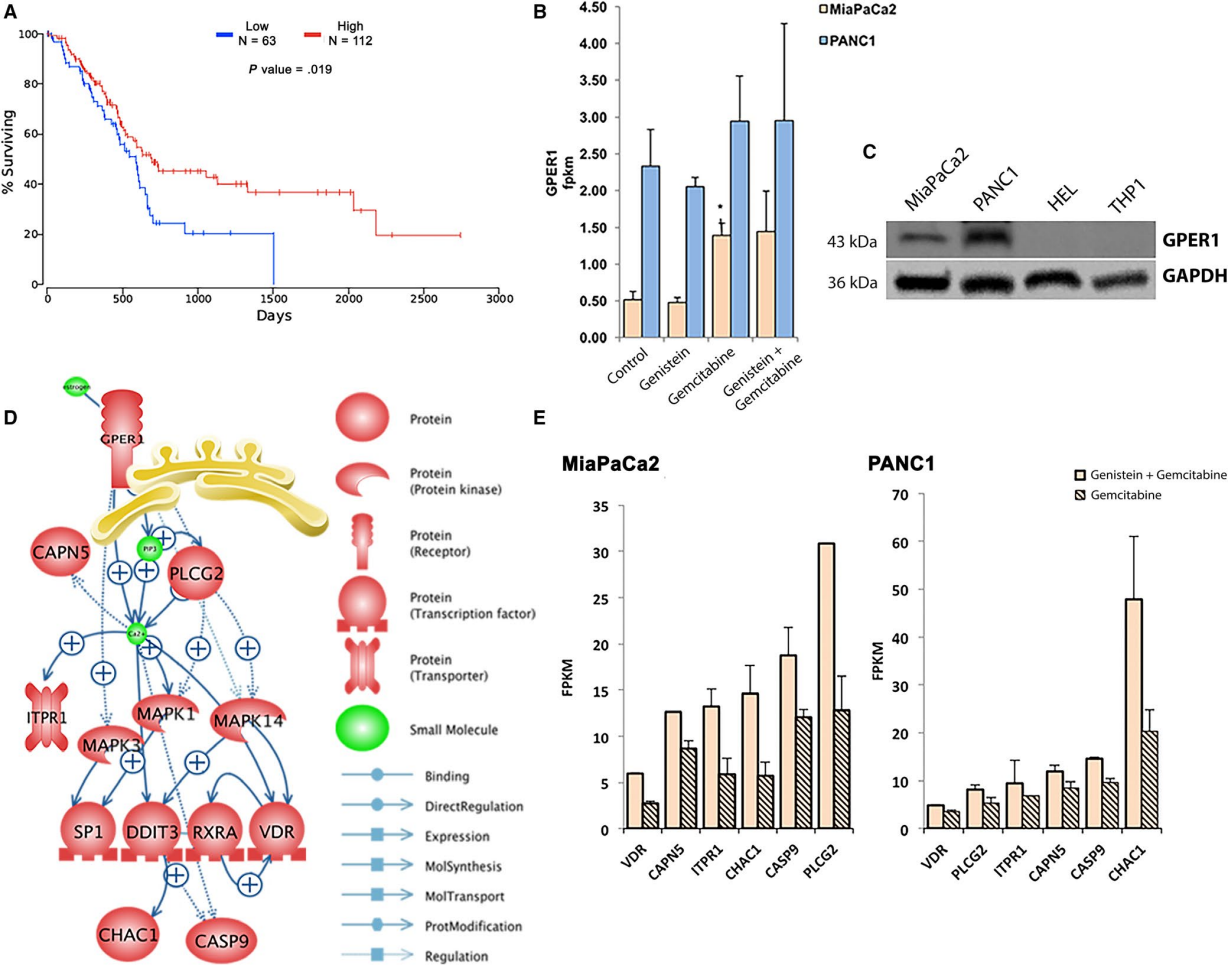
GPER1 as proposed molecular mechanism of genistein in pancreatic cancer cells. A, A higher expression of GPER1 mRNA (>68 RSEM) is correlated with better overall survival (log‐rank test, *P* = .019) in the TCGA cohort of pancreatic cancer patients (n = 175). B, GPER1 mRNA and (C) protein is expressed in both MiaPaCa2 and PANC1, and higher in PANC1. Protein is indicated at approximately 43 kDa, the erythroleukemia HEL and leukemic monocyte THP1 cell lines that have no mRNA evidence of GPER1 (per the http://www.proteinatlas.org) were used as negative controls, and GAPDH was used as a loading control. D, Proposed molecular mechanism of genistein includes activation of the GPER1 and downstream proposed targets, based on RNA‐seq analysis of treated MiaPaCa2 and PANC1 cells. E, Expression (FPKM values) of proposed genistein‐targets upregulated upon co‐treatment with genistein compared to gemcitabine monotreatment (FDR < 0.05) in MiaPaCa2 (left) and PANC1 (right)

Although GPER1 expression was not significantly associated with the response to AXP107‐11‐gemcitabine combination in the PDX ex vivo cultures (Figure 2E), it was expressed 2.6 times higher in sensitive compared to resistant PDXs. GPER1 has not previously been reported to have a role in PDAC, but our RNA‐Seq data indicate its activation. GPER1 is known to affect calcium influx into cytoplasm,^29^ which in turn can control apoptosis,^30^ and both these functions were identified in our analysis. We explored whether GPER1 is expressed in clinical specimens of pancreatic cancer and if it is related to survival. We used the OncoLnc tool^17^ to measure COX coefficients and patient survival of TCGA pancreatic cancer cohort (n = 175). We found that GPER1 is expressed in a proportion of clinical samples of PDAC (64% has >1 FPKM), and that higher expression is linked to better survival in PDAC patients (Figure 4A, top 64% vs low 36%, log rank *P*‐value = .019). This was significant also when comparing lower and upper groups of similar size (low 36% vs high 36%, *P* = .04). We confirmed GPER1 expression in our cell lines at both mRNA and protein levels using qPCR and western blot (Figure 4B,C). We then connected the genistein‐induced gene expression and overrepresented subnetworks with literature mining using the Pathway Studio's software. A model illustrating the generated mechanism of action in pancreatic cancer is illustrated in Figure 4D. This suggested that genistein activates GPER1, which in turn impacts MAPK signaling with subsequent activation of downstream transcription factors (including VDR, RXRA, SP1, DDIT3) and apoptotic signaling (including CASP9). Regulations of indicated key members upon co‐treatment (per RNA‐Seq) is shown in Figure 4E. Based on this data, we propose that genistein can activate GPER1 in pancreatic cells and that this enhances the antiproliferative, apoptotic, and chemoenhancing functions of genistein.

### 3.6 Verification of GPER1 function in pancreatic cancer cells

**Figure 5:**
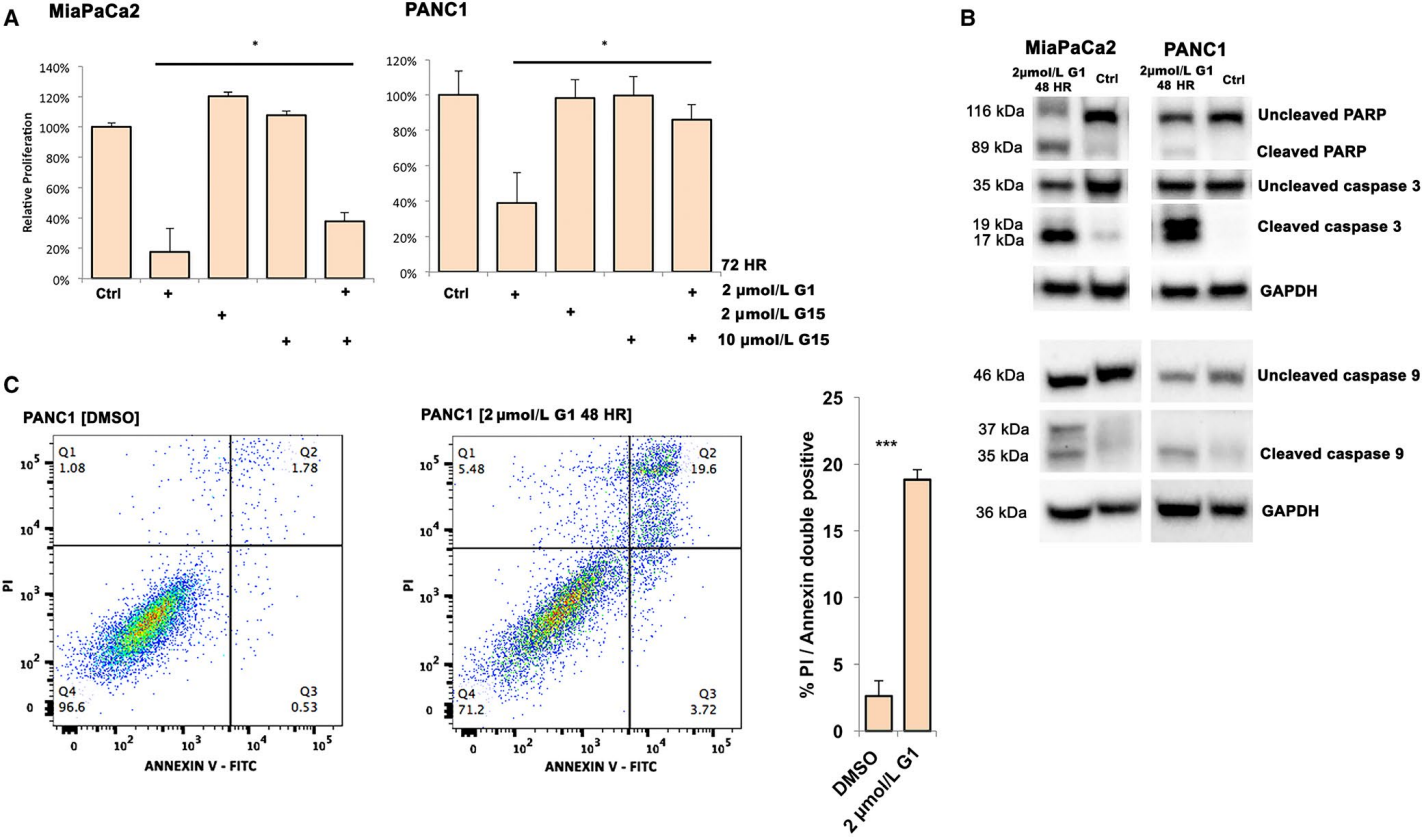
GPER1 mediates anti‐proliferative effects in pancreatic cancer. A, GPER1 agonist (G1, 2 μmol/L) reduces cell proliferation of both cell lines (MTT assay, 72 h), which is attenuated upon addition of GPER1 antagonist (G15). B, G1 induces PARP, caspase 3, and caspase 9 cleavage in both MiaPaCa2 and PANC1. C, Propidium iodide (PI) and Annexin V staining demonstrate apoptosis in PANC1 cells after G1 treatment (2 μmol/L, 48 h). Error bar represents SEM and unpaired two‐tailed *t* test was used to test significance (*P < .05, **P < .01, ***P < .001)

In order to test our hypothesis that GPER1 mediates some or all of the anti‐proliferative and chemoenhancing effects of genistein, we performed experiments with selective GPER1 agonist and antagonists. The selective agonist G1 has been reported to not bind ERα, ERβ, nor 25 other G‐protein coupled receptors at concentrations below 10 μmol/L,^31^ and not induce response in *Gper* knock out mice.^32^ We treated both MiaPaCa2 and PANC1 with different concentrations of G1 for 48 and 72 hours followed by proliferation assay (Figure S2K). A significant reduction of proliferation was observed at 0.5 or 1 μmol/L G1 (72 hours). Combining G1 treatment with GPER1 antagonist G15 could partially (MiaPaCa2) or fully (PANC1) restore cell proliferation (Figure 5A), and G1 enhanced efficacy of gemcitabine in both cell lines (Figure S2L). We corroborated that PARP, caspase 3 (CASP3), and caspase 9 (CASP9) cleavage was activated by G1 treatment (2 μmol/L, 48 hours) in both cell lines (Figure 5B), and FACS analysis demonstrated an 18‐fold increase of apoptosis in PANC1 (Figure 5C). In conclusion, GPER1‐selective ligand mimicked and GPER1 antagonist could block these activities, supporting an antitumorigenic role for GPER1 in PDAC.

## 4 DISCUSSION

Preclinical studies have indicated that genistein have chemoenhancing effects but its efficacy has essentially been unexplored and its precise mechanism of action in PDAC is not known.^13^ Here, we have validated the anti‐proliferative and chemoenhancing properties of genistein and its derivate AXP107‐11 in human PDAC cell lines and explored its efficacy on patient‐derived tumors ex vivo and in vivo. We have further dissected corresponding GPER1‐mediated mechanisms, showed that a higher expression of GPER1 in PDAC is correlated to improved survival in patients, and validated GPER1 activity in PDAC cell lines by using selective agonist and antagonist accompanied by functional assays and marker analysis. Thus, we propose that GPER1 should be clinically evaluated as a potential therapeutic target in PDAC.

GPER1 is a trans‐membrane G protein‐coupled receptor that was cloned in 1997,^33^ but not established as an membrane‐bound estrogen receptor until 2005.^34^ Studies of *Gper1* knockout mice has indicated that GPER1 has a metabolic role in the in vivo pancreas,^35^ but it has previously not been linked to pancreatic cancer. Rather, reports have indicated a role for another estrogen receptor, ERβ, in PDAC.^36^, ^37^ However, the studies which determined that ERβ is expressed in PDAC used antibodies which have been recognized as problematic.^28^ Current TCGA data, based on unbiased large‐scale RNA‐Seq of clinical tumor specimens, do not support the expression of ERβ in PDAC. Out of 176 PDAC cases analyzed, the average level of ERβ was a mere 0.1 FPKM (with no case over 0.6 FPKM). As comparison, other transcription factors are expressed significantly more (eg MYC up to 121 FPKM, and nuclear receptors VDR and NR4A1 up to 23 and 101 FPKM, respectively). GPER1 is expressed in PDAC at considerably higher levels (up to 127 FPKM) than ERβ.

GPER1 has been implicated in cell proliferation in various other tumors, and its ligand was recently demonstrated to be efficacious for treatment of melanoma in mice models.^38^ Genistein activates GPER1 with an EC_50_ of 133 nmol/L.^39^ We note that GPER1 agonists (G1, genistein, AXP107‐11) were more efficacious in MiaPaCa2 than in PANC1 in our study, although GPER1 was expressed at higher levels in PANC1. However, the effect was completely inhibited by antagonist (G15) in PANC1 while this was not the case in MiaPaCa2. It is possible that additional pathways play a role in MiaPaCa2.

Genistein has specific and potent activities by binding to estrogen receptors 40 at concentrations of 1 nmol/L to 1 μmol/L.^41^ At high concentration (>20‐60 μmol/L), genistein can have general cytotoxic effects,^42^ including cell membrane protein perturbations.^43^ In our study, we noted potent chemoenhancing effects at low concentrations (1 μmol/L), which were also corroborated in vivo (AXP107‐11 substance). Although higher doses were used for ex vivo (organotypic cultures) and in vivo (PDX models) due to methodological considerations, ie measuring effects in a shorter time frame than in the clinical setting, this still enables prediction to anticancer drugs in PDAC patients.^21^ While additional mechanisms cannot be excluded, and it is possible that our RNA‐Seq analysis of treated cell lines and ex vivo analyses which used higher concentrations also picked up unspecific effects, our data do identify GPER1 as a main mechanism. We noted that gemcitabine itself upregulated GPER1, which in turn would enable its increased activation if treatment is combined with its ligand (AXP107‐11). We can corroborate impact of GPER1 in cell lines by selective agonist at lower doses (1‐2 μmol/L) and inhibit the effect using antagonist. Further studies should be performed to delineate the impact of reduced proliferation vs cell killing. While we noted significant reduction of tumor growth in vivo, we did not observe significant tumor regression. As treated pancreatic tumors can retain mass due to necrotic cells and dense fibrotic stroma, tumor regression (in the timeframe analyzed) may not be required for clinical impact. More research is however needed to determine whether a clinical impact would be truly significant.

Our combined data support a general clinical evaluation of GPER1 and its ligands as chemoenhancing modifiers in the pharmacological targeting of PDAC.^12^ In this context, it is worth pointing out that PDAC is genetically a highly complex disease^44^ and the potential therapeutic advantages and disadvantages with selective GPER1 agonists and antagonists are complex and not fully understood.^45^ One major limitation of pancreatic cancer therapy is inadequate drug penetration due to high levels of desmoplasia. Our in vivo experiments did not fully capture this clinical presentation, and it is not clear if the proposed GPER1 treatment would overcome this obstacle. However, a recent study which used tamoxifen, which also acts as a GPER1 agonist, in a mouse model of pancreatic cancer, found that this could modify the microenvironment and reduce desmoplasia, inflammation, and immune suppression in PDAC.^46^

As genistein has poor physiochemical characteristics along with low oral bioavailability, a pharmaceutical comprehensive screen for novel crystalline forms of genistein has been performed. Several novel patentable crystalline forms of genistein with improved solubility and oral bioavailability were identified and the genistein sodium salt dehydrate AXP107‐11 was developed as a clinical candidate.^13^ While it is currently not yet experimentally established that AXP107‐11 exactly mimics genistein in the cell, the 3D crystal structure^12^ in combination with our experiments here, show that it has similar functional effects. The pharmacokinetic properties and toxicity of AXP107‐11 in combination with gemcitabine have been evaluated in a phase Ib clinical study in patients with inoperable PDAC.^13^ It was concluded that AXP107‐11 treatment resulted in higher blood concentrations compared to the native form of genistein and was found to be safe and well tolerated.^13^ Although the observed time to progression and median overall survival with the combination treatment was encouraging, it could not be statistically confirmed. Here, we evaluated the efficacy of AXP107‐11 given alone or in combination with gemcitabine in ex vivo cultures of PDX tumors, along with in vivo corroboration and identification of the transcriptomic fingerprint required to elicit an effect in response to AXP107‐11. The ultimate goal of this undertaking was to develop a pharmacogenomics‐based selection of patients likely to respond to AXP107‐11 therapy in combination with gemcitabine. We conclude that such co‐treatment has a synergistic chemoenhancing effect and we identified a 41‐gene signature that may differentiate between responsive tumors. This signature included genes involved in similar pathways as our in vitro‐determined molecular mechanism. Our data are thus encouraging for continued clinical evaluation and development of AXP107‐11 for PDAC.

Finally, the transcriptome data we provide could prove a valuable resource for studies attempting to establish the molecular mechanism of compounds that are intended to overcome the resistance to gemcitabine. We identified that short‐term (24 hours) gemcitabine monotherapy activated several pathways that have been linked to chemoresistance in PDAC. These include P13/AKT1,^47^ TGFβ1,^48^ and TNFα/NFκB^8^ signaling pathways. Activation of the latter has been reported to generate gemcitabine resistance in PDAC cell lines through an NFκB‐HIF1α‐CXCR4 positive feedback loop^49^ and/or by increasing MUC4.^50^ Overexpression of mucins is implicated in chemoresistance in multiple tumors, including MUC1 in pancreatic cancers.^26^ Thus, we also identified gene pathways whereby gemcitabine treatment itself directly enables resistance mechanisms.

In conclusion, we present data delineating the mechanism of action for genistein, and the chemically related clinical candidate AXP107‐11, as chemoenhancers in PDAC both in vitro and in vivo. We also propose a transcriptomic signature that is associated with a positive response in human‐derived pancreatic tumors. Taken together, our study provides support for a pharmacogenomics‐based personalized treatment resulting in a potentially more efficient management of a subset of PDAC patients.

## CONFLICT OF INTEREST

Anders Berkenstam was founder of and previous consultant to Axcentua Pharmaceuticals.

## Data Availability

Data is uploaded under GEO accession number [http://www.ncbi.nlm.nih.gov/geo/query/acc.cgi?acc=GSE97766].
