# Supplementary figures and images for: Clinical candidate and genistein analogue AXP107‐11 has chemoenhancing functions in pancreatic adenocarcinoma through G protein‐coupled estrogen receptor signaling
Source: Cancer Med. 2019 Sep 30;8(18):7705–19. doi: 10.1002/cam4.2581 (PMC6912054; doi:10.1002/cam4.2581)

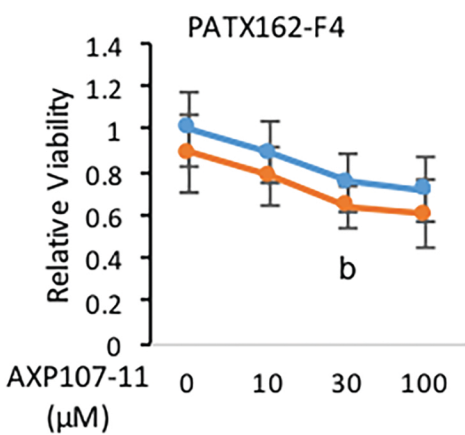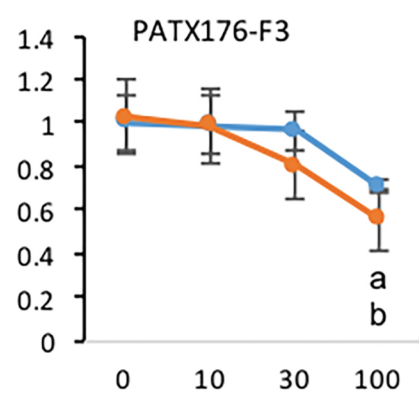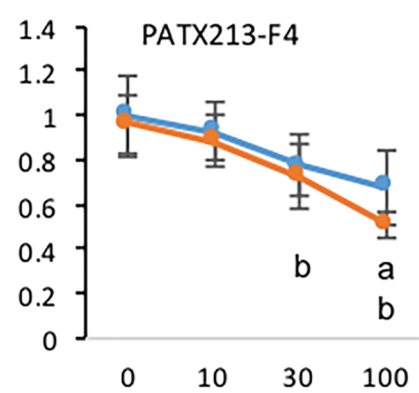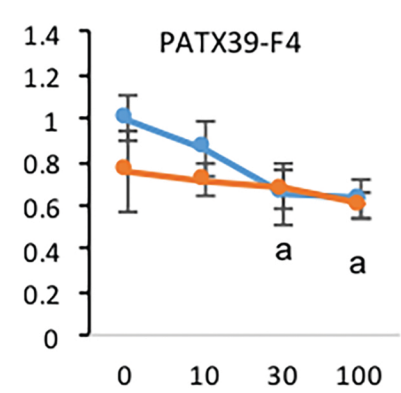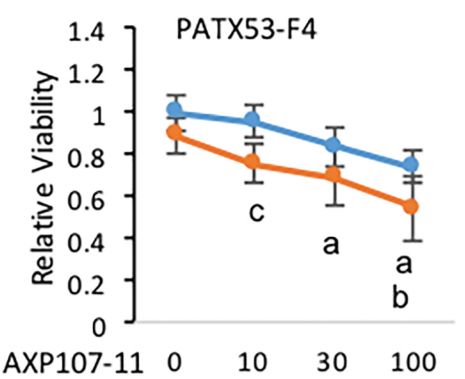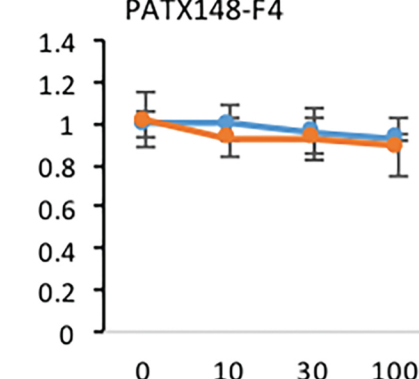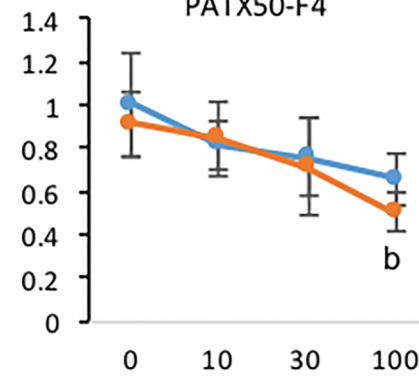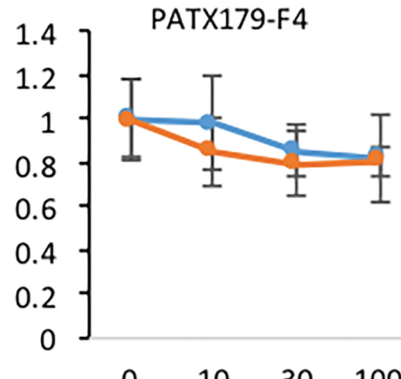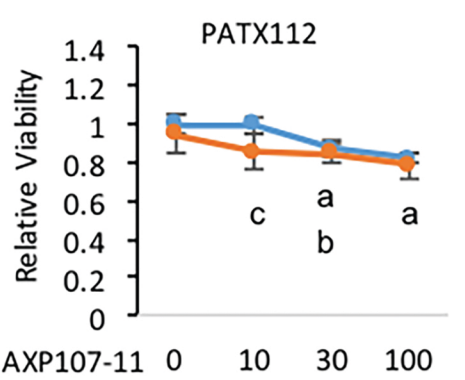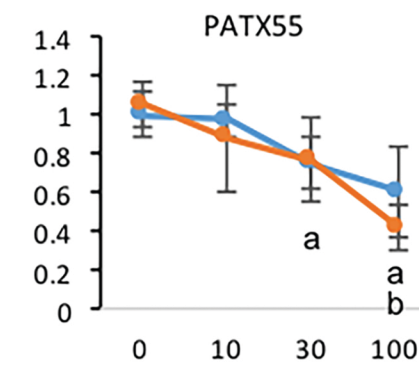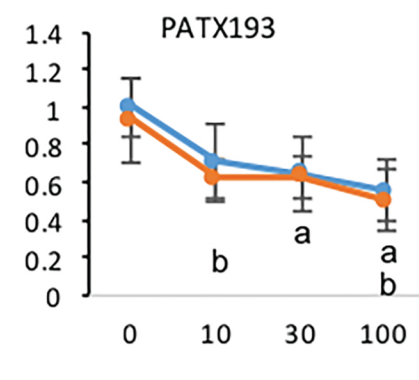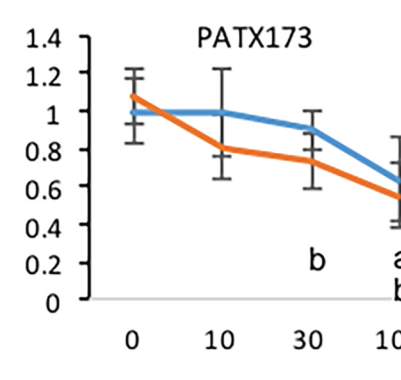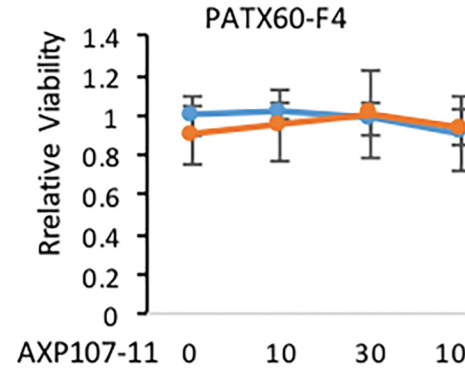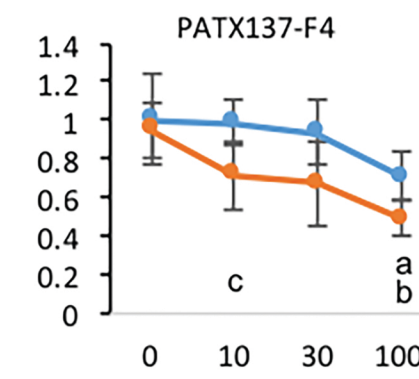

— Vehicle (DMSO)  
— Gemcitabine (10 μM)

Supplement: Supplementary file 1 [file CAM4-8-7705-s001.pdf]

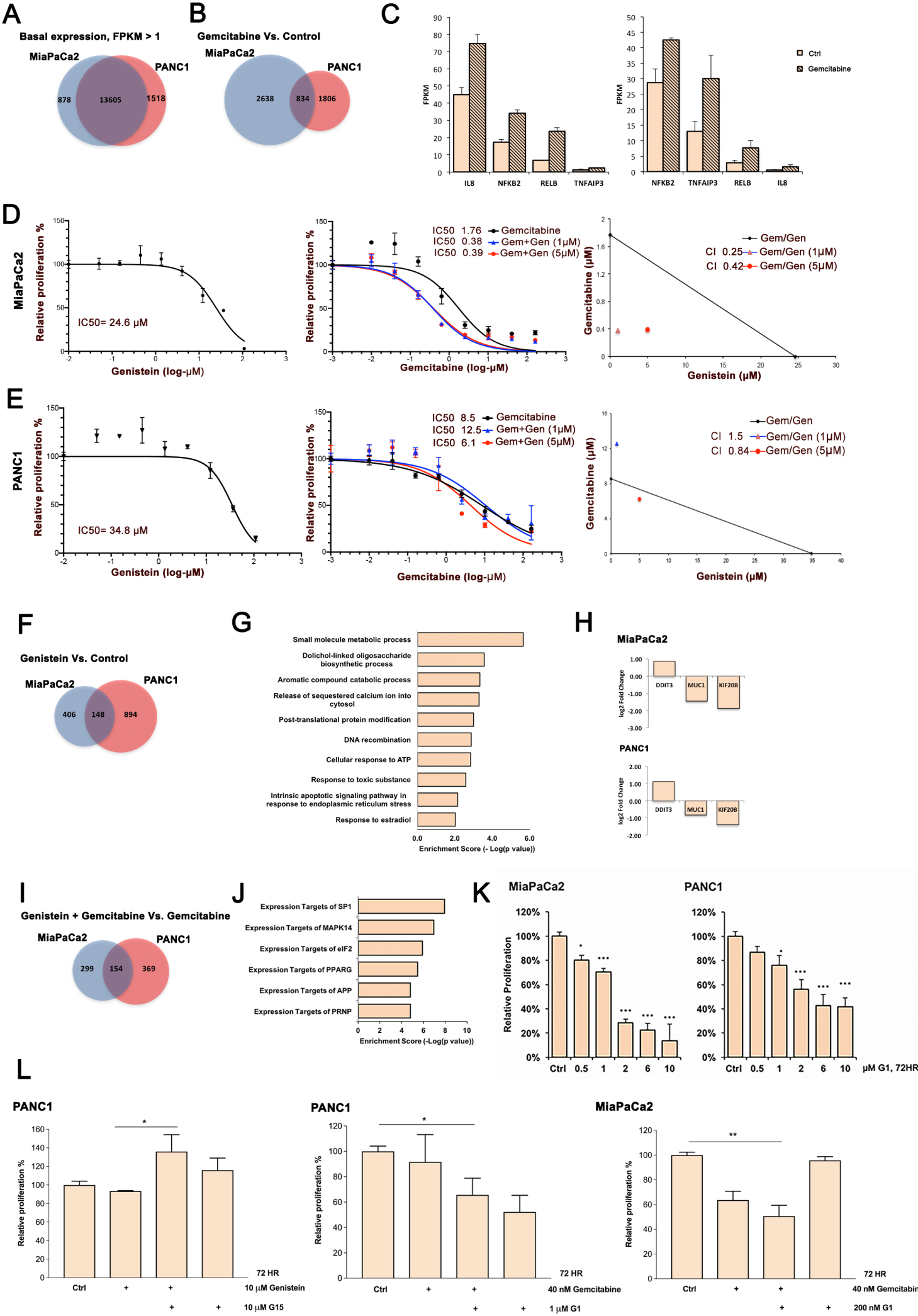

Supplement: Supplementary file 2 [file CAM4-8-7705-s002.pdf]

B

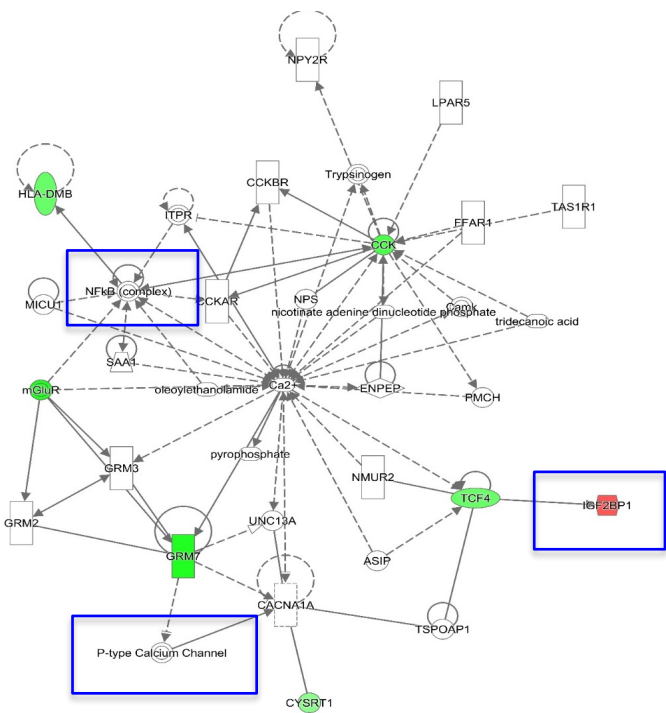

Supplement: Supplementary file 3 [file CAM4-8-7705-s003.pdf]
